# Editorial: Alternatives to Combat Bacterial Infections

**DOI:** 10.3389/fmicb.2022.909866

**Published:** 2022-05-04

**Authors:** Rodolfo García-Contreras, Mariano Martínez-Vázquez, Bertha González-Pedrajo, Israel Castillo-Juárez

**Affiliations:** ^1^Departamento de Microbiología y Parasitología, Facultad de Medicina, Universidad Nacional Autónoma de México, Ciudad de México, Mexico; ^2^Departamento de Productos Naturales, Instituto de Química, Universidad Nacional Autónoma de México, Ciudad de México, Mexico; ^3^Departamento de Genética Molecular, Instituto de Fisiología Celular, Universidad Nacional Autónoma de México, Ciudad de México, Mexico; ^4^Laboratorio de Fitoquímica, Posgrado de Botánica, Colegio de Postgraduados, Texcoco, Mexico

**Keywords:** bacteriophage, drug repurposing, nanoparticles, antimicrobial peptides, quorum quenching, biofilms, probiotics

In 2019, infections due to multidrug resistant (MDR) bacteria caused 1.27 million deaths (Collaborators, 2022), and the projections indicate that if the current prevalence of antibiotic resistance continues increasing and if no new effective therapies are implemented, in the year 2050, there will be around 10 million deaths worldwide because of these infections (de Kraker et al., 2016). Hence, the World Health Organization has urged for the development of new antibiotics or other alternatives to combat MDR bacteria. Of particular importance are those bacteria belonging to the ESKAPE group since they are responsible for difficult or impossible to treat nosocomial infections related to multidrug and pan drug resistance. Many other bacteria such as members of the *Mycobacterium* genus or the Enterobacteriaceae family are also a serious threat to global health. In this Research Topic, 30 excellent articles dealing with different proposals of alternatives to combat bacterial infections are included.

## Bacteriophages and Phage Components

Bacteriophages are viruses that replicate and kill bacteria and although they have been used in some eastern European countries, their use in western medicine remains underutilized due the availability and efficacy of antibiotics. However, currently there is renewed enthusiasm, and several researchers and clinical doctors are working in their utilization to treat recalcitrant infections.

In the research of Manohar et al. the effect of bacterial coinfections during pulmonary viral disease was studied, with an emphasis on COVID patients. Viral respiratory infections increase the susceptibility for bacterial infections, which cause epithelial damage and mucociliary clearance impairment. Around 50% of the deaths of infected SARS-COV-2 patients are caused by secondary MDR bacterial infections; hence bacteriophages or phage components like endolysins could reduce the mortality of coinfected patients, since phages are specific, self-replicating, nontoxic to hosts, and can have synergy with antibiotics.

In the review by Kumar et al. an overview of resistance and the role of anthropogenic sources and commercial livestock as generators of resistance on a global scale are discussed. Also, they uncovered some of the main unconventional and novel strategies currently being investigated that have the potential to reduce infections and to prevent the increase in resistance. Some of them are the identification of stem cell-derived antimicrobial peptides, CRISPR-Cas based therapies, nanoantibiotics, fecal transplantation, immunotherapies, microbial therapies (probiotics, postbiotics, and synbiotics), and phages, as well as the inhibition of quorum sensing (QS).

## Drug Repurposing and Combination Therapies

Repurposing drugs for their utilization as antimicrobials could significantly accelerate the implementation of novel antimicrobial therapies that otherwise may take decades to be launched. Gallium nitrate, previously used for hypercalcemia in malignity, was repurposed as an antibacterial agent for treating infections in humans (Goss et al., 2018). Gallium is a non-REDOX mimetic form of Iron 3^+^, which is incorporated into iron-containing enzymes rendering them unable to perform their functions. Gallium also sequesters pyoverdine, the main siderophore of *P. aeruginosa*, decreasing iron uptake. Zemke et al. demonstrated that combining gallium treatment with nitric oxide (NO) had synergistic effects against *P. aeruginosa*, since nitrosative stress induces damage to iron metalloproteins both in aerobic and anaerobic conditions. Furthermore, *P. aeruginosa hitA* mutants resistant to gallium (García-Contreras et al., 2013) were also sensitized by nitrite.

An example that suggests that the combination of drugs prevents resistance to enterococci is the research carried out by Jiang et al. In their study, they analyzed five clinical isolates of *Enterococcus*, identifying that resistance to linezolid was due to mutations in ribosomal proteins, while resistance to fosfomycin in some isolates was due to mutations in *murA*. In these strains, two synergistic combinations of linezolid and fosfomycin were able to suppress the selection of resistant mutants. These results prove that a correct combination of linezolid with fosfomycin favors synergy and reduces resistance induction.

Li et al. used high-throughput screening and drug repurposing to develop combined therapy of a tetracyline antibiotic and an AMP to combat *P. aeruginosa*. They showed that this combination had a significant synergistic effect on antibacterial activity as compared to monotherapies. Also, the effectiveness of the combined approach was verified *in vivo* in a cutaneous murine model of infection, in which both the abscess size and inflammation of the skin abscess caused by this pathogen were considerably reduced with the treatment. These data illustrate the relevance of improving the effects of existing antibiotics as an encouraging method to enhance the efficacy of antibacterial treatments.

## Nanoparticles and Bactericidal Compounds

Nanoparticles containing metals such as silver and copper have been shown to be effective antibacterial compounds, in addition they can be used as carriers of bactericidal compounds and their synthesis using extracts from plants is simple and scalable. Cheng et al. synthesized silver nanoparticles using *Canna indica* L, Cosmo bipinnata Cav., and Lantana camara flower aqueous extracts and tested them against the phytopathogen Ralstonia solanaceum. The functionalized silver nanoparticles were characterized by UV-visible spectroscopy, FTIR, and XRD. The results showed that *L. camara* silver nanoparticles had the smallest particle size and the highest effect on bacterial growth, biofilm formation, swimming motility, efflux of nucleic acid, cell death, cell membrane damage, and ROS generation. The silver nanoparticles synthesized with the *L. camara, C. bipinnata*, and *C*. *indica* flower aqueous extracts could be used as an efficient and environmentally friendly antibacterial agent against *R. solanaceum*.

The bactericidal actions of Azadirachta indica are well known (Herrera-Calderon et al., 2019). As a new proposal to fight bacterial infections, a hydrogel constituted by silver particles synthesized from an aqueous extract of *A. indica* loaded on PF127, a biocompatible-biodegradable polymer, was used. *A. indica* silver nanoparticles (AI-AgNPs) are effective antibacterial compounds, showing higher antioxidant effects than leaf extracts alone. Additionally, the AI-AgNPs did not show toxicity on *Drosophila melanogaster*. The application of the AI-AgNPs-PF127 hydrogel improved the wound contraction rate in mice. The synergistic interactions between the Ag^+^ ions and phytochemicals in the extract formed stable bioactive AI-AgNP molecules that displayed better antibacterial efficacy than the AI extract (Chinnasamy et al.).

Enterococci, normal microbiota in the gastrointestinal tract of humans, can infect wounds, the bloodstream, and urinary tract. *Enterococcus faecalis* are the cause of most infections which are difficult to treat due to resistance against antibiotics. Xiong et al. evaluated the antibacterial properties of telithromycin, a semi-synthetic derivative of erythromycin, against a panel of 280 *E. faecalis* and 122 *E. faecium* isolates, finding good antibacterial activity. Moreover, telithromycin also showed antibiofilm activity at growth sub inhibitory doses, and at higher doses, combined with ampicillin, it was able to kill biofilm cells and reduce established biofilms. This is a promising alternative for the successful treatment of enterococci infections.

## Antimicrobial Peptides and Compounds

One of the most promising alternatives for combating MDR bacterial infections are antimicrobial peptides (AMPs), which are part of the innate immune response of mammals and other animals. AMPs disrupt the membrane of pathogens and have immunomodulatory effects, hence, their utilization in antibacterial activities is very attractive.

*Staphylococcus aureus* is a Gram-positive bacterium of the ESKAPE group. In their work, Zhang X et al. evaluated the mechanism of the novel antimicrobial agent isopropoxy benzene guanidine (IBG) against *S. aureus*. IBG has a low MIC against several *S. aureus* strains, including MRSA. The IBG mechanism of action involves membrane disruption and lysis. Remarkably, the rate of resistance selection against this compound was very low, compared to the resistance selection against ciprofloxacin. Moreover, IBG increased the survival of mice with septicemia approximately 4-fold relative to untreated mice.

AMPs are less prone than regular antibiotics to induce resistance; however, resistance mechanisms against them exist. In their review work, Assoni et al. discuss the known mechanisms of AMP resistance in Gram-positive bacteria. These include modifications of the membrane and cell wall, reduction of the negative charge of the bacterial surface, decrease in peptide affinity, efflux pumps that expel the peptides, variation in the capsule polysaccharide, and, in some cases, AMP sequestration and cleavage by proteases.

The implementation of AMPs in the clinic is challenging because of their high development and production costs, cytotoxicity, reduced activity in clinically relevant environments, and bacterial resistance. Furthermore, AMPs acting on membranes are not entirely selective for microbes. Several strategies have been designed to overcome these challenges, such as the preparation of ultra-short/truncated AMPs, delivery systems, chemical modifications, and the careful selection of a counter-ion in the last step of AMP synthesis. Nonetheless, several AMPs in clinical trials have failed. The following practical strategies should be considered in future clinical testing: defining optimal doses and administration of regimens to reduce cytotoxicity, including bacterial resistance development as a primary outcome parameter in the trials, the bioavailability and efficacy of AMPs can be improved using delivery systems, and combining AMPs with antibiotics or other compounds might improve antimicrobial effects (Dijksteel et al.).

*Bacillus* produce several polypeptide antibiotics. Some of them like bacitracin, gramicidin S, polymyxin, and tyrotricidin are used in the clinic (Yilmaz et al., 2006). In the paper of Lin et al., three *Bacillus* strains with excellent antimicrobial properties named JFL21, LQG17, and LQG36, were isolated and identified to be related to *B. amyloliquefaciens, B. subtilis*, and *B. halotolerans*, respectively. The antimicrobial substances produced by *B. amyloliquefaciens* JFL21 had low toxicity to most probiotics but exhibited strong and extensive antimicrobial activities against MDR foodborne pathogens. The FITR, HPLC, and MALDI-TOF MS analysis revealed that the partially purified anti-JFL21 substance comprises multiple lipopeptides of the surfactin, fengycin, and iturin families. Fengycins were also highly stable against a variety of enzymes, chemical reagents, and extreme conditions.

Another work on the topic of dealing with AMPs was that of Mazumdar et al., in which two peptides derived from a bacteriocin of *Lactobacillus casei* were designed, synthesized, and characterized. Remarkably, both peptides showed low MICs (10 to 30 μg/ml) against *E. coli* and several Gram-positive pathogenic bacteria, including methicillin and vancomycin-resistant *S. aureus* (MRSA and VSRA) and *Enterococcus faecalis* resistant to vancomycin. The peptides caused damage to the bacterial cell wall leading to leakage of intracellular content and bacterial death, and were also effective against clinical strains isolated from wounds. In contrast, they presented low toxicity against mammary glands and epithelial cells and were effective at promoting bacterial clearance *in vivo* and recovery of mice infected with VRSA, making them very promising for the eventual treatment of animal or human infections.

## Probiotics

Probiotics are beneficial microorganisms that promote intestinal health when consumed, due to their regulatory effects in the microbiota and metabolism. In their work, Zeng et al. isolated *Bacillus* proteolyticus (Z1 and Z2), *Bacillus amyloliquefaciens* (J), and *Bacillus subtilis* (K), from yak intestinal micro-ecosystems. Their probiotic potential was evaluated. Antioxidant activity examinations indicated that Z1 had the most elevated DPPH and hydroxyl radical scavenging activities, whereas Z2 had higher reducing power and inhibited lipid peroxidation. All strains were antagonistic to three indicator pathogens, *E. coli, S. aureus*, and *S. enteritidis*. These isolates also had a higher hydrophobicity, auto-aggregation, and acid and bile tolerance, all of which permitted survival and kept dangerous bacteria out of the host intestine. Importantly, all strains could be considered safe because of their antibiotic susceptibility and lack of hemolysis. This is the first study to show that *B. proteolyticus* and *B. amyloliquefaciens* isolated from yaks have a probiotic potential.

## Inhibition of Quorum Sensing and Biofilm Formation

Biofilms are the preferred lifestyle of bacteria and are involved in approximately 60% of infections; they shield bacteria from several stressors, including antibiotics, increasing their tolerance up to 1,000-fold. Part of the maturation of biofilms is mediated by QS that allows bacteria to sense their population density and change gene expression accordingly. In their review, Zhou et al. described the regulatory circuits of QS in Gram-positive bacteria, and the approaches to interfere with them that led to biofilm inhibition, degradation of signal molecules, receptor blocking, and inhibition of QS cascades. They identified several approaches for the discovery of more QS-interfering agents and discussed the application of QS inhibitors in the clinic, food industry, and water treatment strategies.

Sethupathy et al. reported the activity of 51 indole derivatives on the inhibition of QS and biofilm formation in *Serratia marcescens*, of which 6-fluoroindole and 7-methylindole stood out for their ability to reduce virulence, QS, and biofilms.

Valliammai et al. identified a strong anti-virulence activity of thymol on methicillin-resistant *Staphylococcus aureus*. Also, thymol exhibited SarA-dependent anti-biofilm activity reducing its ability to adhere to glass and metal surfaces. Similarly, it improved the bactericidal, biofilm, and persistent cell eradication efficacy of rifampicin.

An et al. reviewed biological and computational methods for designing mechanism-informed anti-biofilm agents. They postulated the use of omics analyses to provide a biological approach to the complex processes of biofilm formation, uncovering many potential protein targets and pathways required for biofilm formation in a variety of species. To find modulators for these targets, they completed a virtual screening of large databases of molecules before experimental validation. Another approach to identify novel anti-biofilm agents is through machine learning, where a computational model is trained using a collection of known antibiofilm and non-antibiofilm molecules and then is used to find potential previously unknown antibiofilm compounds. Finally, these new agents must be evaluated in biologically accurate biofilm models including *in vitro, in vivo*, and organoid-on-a-chip models.

*A. baumannii* has a QS system (*abaI/abaR*) mediated by acyl-homoserine-lactones (AHLs) and several quorum quenching (QQ) enzymes. Nevertheless, the roles of this complex network in the control of the expression of surface-associated motility and biofilms are not evident. Therefore, the effect of the mutation of the AHL synthase *abaI* and the exogenous addition of the QQ enzyme Aii20J on surface-associated motility and biofilm formation in *A. baumannii* ATCCR 17978TM was studied. The results showed that extracellular DNA is a main component of the extracellular matrix in *A. baumannii* biofilms since the QQ enzyme Aii20J and DNases reduced biofilm formation in all tested strains. These findings revealed that QQ strategies combined with other enzymes such as DNase can prevent *A. baumannii* colonization and survival on surfaces (Mayer et al.).

Another important health problem affecting millions of people are dental caries, caused by the bacterial metabolism of species such as *Streptococcus mutans*, which catabolize carbohydrates producing acid. The expression of acid resistance mechanisms is fundamental for the survival of these bacteria, among them, F0F1-ATPase expels protons from the cytosol. Zhang M et al. evaluated bedaquiline, an inhibitor of this enzyme used for MDR tuberculosis, against *S. mutants* and related bacteria. They found that bedaquiline had bacteriostatic activity and an antibiofilm effect at acidic pH. It also showed low cytotoxicity, selectively attacking caries promoting bacteria in acidic environments.

Sateriale et al. evaluated the effect of hydroethanolic extracts (rich in polyphenols) of myrtle leaf and dry pomegranate fruit peel, alone and in combination, on biofilm formation of *S. mutans, S. oralis, S. mitis*, and *Rhotia dentocariosa* oral isolates, finding effective concentrations in the range of 10 to 40 mg/ml. Moreover, at higher concentrations the extracts eradicated preformed single and multispecies biofilms. Although oral biofilm such as dental plaque is complex, this study encourages further investigation into the implementation of polyphenolic extracts for the inhibition of dental plaque.

## Photodynamic Therapy

One novel approach to combat MDR bacterial infections is the utilization of photosensitizer compounds that react with light-producing reactive oxygen species, killing bacteria. In their work, Maldonado-Carmona et al. encapsulated a porphyrin derivate into acetylated lignin nanoparticles, and characterized the physicochemical characteristics of the particles. They showed that the particles were stable in a wide pH range (4–10) and retained their activity after 2 months. The encapsulated compound generated an oxygen singlet and was effective for killing both Gram-negative and positive bacteria including *P. aeruginosa* and *S. aureus*, when exposed to white light, targeting the cell wall. The utilization of these kinds of antimicrobials in topical infections could be effective to treat MDR bacteria.

In addition to the use of exogenous photosensitizers, violet and blue light directly excite endogenous molecules present in bacteria allowing the production of reactive oxygen species upon contact with O_2_. Based on this phenomenon, Hoenes et al. evaluated the effect of this radiation in ESKAPE bacteria. Light irradiation decreased the colony-forming units of all tested bacteria in a dose response manner; interestingly the most sensitive bacteria were *Acinetobacter*, the number one critical bacteria for which it is urgent to develop new antimicrobials.

Another work dealing with the application of photodynamic therapy for the treatment of bacterial infections was done by Zhao et al. They used protoporphyrin IX-methyl ethylenediamine (PPIX-MED) as a photosensitizer which produced an oxygen singlet, and was first tested *in vitro* against clinical isolates of *E. coli, P. aeruginosa*, and MRSA *S. aureus*. The compound had MIC100 and bactericidal concentrations in the micromolar range. Later, its effect alone and in combination with ceftriaxone for treating mixed infections in burned rats was tested. The treatment, including the antibiotic plus PPIX-MED and light, promoted faster healing of the wounds than treatments with PPIX-MED or ceftriaxone alone, resulting in a decrease in bacterial counts in the wounds and blood, neovascularization, and wound.

Beyond their applications for human and animal infections, photosensitizers can also be used to treat plant diseases, such as citrus canker, produced by *Xanthomonas citri subsp. Citri* (Xcc) which damages citrus plants and economically affects the citrus industry. Jiang et al. used photodynamic therapy to kill this bacterial pathogen. They developed a stable photosensitizer complex (PSC) which, when activated by sunlight, produces reactive oxygen species that kill bacteria. This antibacterial agent was shown to be much more potent than the copper salts currently used to control it. It also showed low toxicity on citrus leaves when using a solar simulator. Therefore, treatment with this PSC is an effective method to eradicate *Xcc* and a promising strategy to control citrus canker.

Beyond small molecules, enzymes, and bacteriophages, the utilization of certain host cells is also a robust alternative to combat MDR infections. In their review work, Russell et al. explain that mesenchymal stromal cells (MSCs) have several direct and indirect mechanisms that make them suitable for this purpose. Among the direct mechanisms, these cells are producers of AMPs, indoleamine 2,3-dioxygenase, and nitric oxide, and result in phagocytosis. Indirect mechanisms include recruitment of immune cells, macrophage stimulation, and phenotype modulation. Then they discuss what is known about the interaction of MSCs with antimicrobials, and provide current evidence supporting their beneficial effects in veterinary medicine.

The elucidation of the resistance mechanisms against antibiotics in MDR bacteria helped to identify therapeutic targets for the development of antibacterials. In this regard, Yang et al. evaluated the role of the cytosolic glucosaminidase NagZ in the overexpression of the chromosomally encoded β-lactamase AmpC in *Enterobacter cloacae*, using clinical isolates. The findings revealed a higher expression of *nagZ* and *ampC* in isolates resistant to one 3rd or 4th generation cephalosporin, relative to sensitive ones, correlating with higher β-lactamase activity. Moreover, deletion of *nagZ* in a resistant isolate decreased its β-lactam resistance, while ectopic expression of *nagZ* in a sensitive isolate, rendered it resistant. Their work indicates that the mechanism of the NagZ-mediated *ampC* overexpression is due the production of 1,6-anhydromuropeptides by NagZ, which activates AmpR).

In addition to finding new drugs for the treatment of MDR bacterial infections, it is also important to optimize the administration schemes of currently used antibiotics. Song et al. compared the traditional administration of vancomycin [intermittent i.v. infusion (ITII)] with a newly designed two-step infusion method (OTSI), using pharmacokinetic and pharmacodynamic modeling. Their analysis indicated that the new method outperformed the traditional one for the treatments of several infections including pericarditis, mastitis, bacteremia, and pleura infections, and could be effective to treat infections caused by isolates of bacteria such *E. faecalis, S. aureus, S. epidermidis*, and S. *bovis* with high MICs against vancomycin.

## Novel Antibacterial Mechanisms

Classical antibiotics disrupt processes such as cell wall synthesis, protein synthesis, DNA replication, and membrane integrity, nevertheless many other processes and targets are also essential for bacterial replication or viability and hence could be exploited as novel antibacterial targets. In an interesting work, Chen et al. investigated the details of the antibacterial agent cryptotanshinone (CT), which is a quinone isolated from the plant *Salvia miltiorrhiza*, which has a wide antibacterial effect against Gram-positive bacteria. They demonstrated that it has bacteriostatic activity and that it targets the membrane-causing dissipation of membrane potential. Further experiments showed that CT is a respiratory chain inhibitor, specifically targeting type II NADH:quinone dehydrogenase, a fact that would provide specificity due to the lack of this enzyme in mammalian mitochondria. Another attractive property of CT is that it has synergistic effects with respiratory inhibitors that have different targets, and hence a possible combinatory therapy may be formulated to combat MDR Gram-positive infections.

## Efflux Pumps

Efflux pumps simultaneously confer resistance against several antibiotic classes. In their opinion work, Nazarov et al. discuss the main efflux pump in *Escherichia coli*, the AcrAB-TolC system, and its role in resistance against the novel antimicrobial agent SkQ1, which interferes with bacterial bioenergetics. SkQ1 is effective against Gram-positive bacteria—it is *E. coli*-resistant—since AcrAB-TolC effluxes SkQ1. Regarding other Gram-negative bacteria that have the AcrAB-TolC pump, some of them like *Klebsiella pneumoniae* that have an identity of 91.5% with the pump of *E. coli* are also resistant, while others which have lower identities are sensitive, indicating their AcrAB-TolC pump is unable to efflux SkQ1. Hence, although being phylogenetical homologs, they are not homologous in function and should be considered paralogs.

## Author Contributions

All authors listed have made a substantial, direct, and intellectual contribution to the work and approved it for publication.

## Funding

RG-C research is funded by CONACYT grant CB 2017–2018 number A1-S-8530 and by DGAPA-UNAM grant no. IN214218. BG-P research is funded by DGAPA-UNAM grant no. IN212420 and by CONACYT grant no. 284081. IC-J research is funded by Cátedras-CONACyT program.

## Conflict of Interest

The authors declare that the research was conducted in the absence of any commercial or financial relationships that could be construed as a potential conflict of interest.

## Publisher's Note

All claims expressed in this article are solely those of the authors and do not necessarily represent those of their affiliated organizations, or those of the publisher, the editors and the reviewers. Any product that may be evaluated in this article, or claim that may be made by its manufacturer, is not guaranteed or endorsed by the publisher.
